# CH_3_COOAg with Laccase-like Activity for Differentiation and Detection of Aminoglycoside Antibiotics

**DOI:** 10.3390/bios15090570

**Published:** 2025-09-01

**Authors:** Huan Zhu, Tong-Qing Chai, Jia-Xin Li, Jing-Jing Dai, Lei Xu, Wen-Ling Qin, Feng-Qing Yang

**Affiliations:** 1 School of Chemistry and Chemical Engineering, Chongqing University, Chongqing 401331, China; 202318021131T@stu.cqu.edu.cn (H.Z.); 20175531@cqu.edu.cn (T.-Q.C.); 202218021016@stu.cqu.edu.cn (J.-X.L.); 202318021129T@stu.cqu.edu.cn (J.-J.D.); 20195277@cqu.edu.cn (L.X.); 2 Chongqing Key Laboratory of Natural Product Synthesis and Drug Research, School of Pharmaceutical Sciences, Chongqing University, Chongqing 401331, China

**Keywords:** CH_3_COOAg, laccase-like activity, sensor array, aminoglycoside antibiotics, colorimetric

## Abstract

Aminoglycoside antibiotics (AGs) are widely used in medicine and animal husbandry, but they pose significant risks due to residual toxicity and antibiotic resistance. In this study, a novel chemical sensor based on the laccase-like activity of CH_3_COOAg was developed for the selective detection of AGs. CH_3_COOAg exhibited varying degrees of laccase-like activity in different buffers (MES, HEPES, and NaAc) and H_2_O, and five AGs showed distinct intensities of the inhibitory effect on the laccase-like activity of CH_3_COOA in different buffers and H_2_O. Therefore, a four-channel colorimetric sensor array was constructed in combination with the use of principal component analysis (PCA) and Hierarchical Cluster Analysis (HCA) for the efficient identification of five AGs (0.02–0.3 μM) in environment samples like tap and lake water. In addition, a colorimetric method was developed for kanamycin (KAN) detection in a honey sample with a linear range of 10–100 nM (R^2^ = 0.9977). The method has excellent sensitivity with a limit of detection of 3.99 nM for KAN. This work not only provides a rapid and low-cost detection method for AG monitoring but also provides a reference for the design of non-copper laccase mimics.

## 1. Introduction

Aminoglycoside antibiotics (AGs) are a class of broad-spectrum antimicrobial drugs consisting of an aminosugar molecule linked to an aminocyclic alcohol through a glycosidic bond [1], such as kanamycin (KAN), gentamicin sulfate (GEN), ribostamycin sulfate (RSM), streptomycin (STR), and tobramycin (TOB) [2]. AGs inhibit peptide chain elongation through binding to the 16S rRNA of the 30S ribosomal subunit, thereby blocking protein synthesis and impeding the growth of bacteria [3,4]. Due to their effectiveness in treating infections caused by both Gram-negative and Gram-positive bacteria [5], AGs are widely used in animal husbandry and the medical field [6,7]. However, the excessive or improper use of AGs in livestock production can result in environmental contamination and antibiotic residues in animal-derived foods, including milk, honey, and meat [8]. Humans may ingest excessive amounts of AGs through the food chain, which can result in many adverse effects, such as ototoxicity [9], nephrotoxicity [10], allergic reactions [11], and antibiotic resistance [12]. The maximum residue limits (MRLs) of AGs have been established in many countries and regions [13]. For instance, the European Union (EU) has established MRLs for KAN, including 100 μg/kg for meat, 600 μg/kg for liver, 2500 μg/kg for kidney, and 150 μg/kg for milk [7]. Furthermore, the Community Reference Laboratory in France has set a recommended concentration of 40 μg/kg of streptomycin in honey [14]. Therefore, it is important to establish sensitive methods for monitoring AGs in foods of animal origin to ensure safety and protect human health. Several methods have been developed for the detection of AGs, such as electrochemical methods [15], liquid chromatography coupled with tandem mass spectrometry [16], microbiological detection methods [17], enzyme-linked immunosorbent assays [18], immunochromatographic analysis [19], and fluorescent methods [20]. These methods are often expensive, sophisticated, and time-consuming. In addition to the above approaches, colorimetric methods are also used for AG detection [21,22], which have the advantages of operational simplicity, low cost, and the possibility of detecting/quantifying pollutants by the naked eye [23].

Usually, colorimetric sensors struggle to selectively detect specific classes of antibiotics and can only detect one antibiotic due to their lack of ability to detect multiple targets. Unlike conventional “lock-and-key” methods, sensor arrays based on the “electronic nose/tongue” strategy can simultaneously detect and discriminate between multiple target analytes with similar structural properties [24,25,26,27]. The sensor array is composed of different sensor units, each of which can generate different signal intensities in response to a range of analytes. Multiple sensors collectively create a unique fingerprint signal for the same analyte [28,29,30]. Two pattern recognition methods, principal component analysis (PCA) and Hierarchical Cluster Analysis (HCA), are used to analyze the data, which can achieve the molecular recognition of specific substances [31,32]. Sensor arrays have the advantages of fast response, high sensitivity, low cost, and rich output signals [33]. Currently, some colorimetric sensor arrays are used for antibiotic analysis [2,34,35], but most of them focus on the detection of tetracycline antibiotics. Therefore, it is significant to develop novel colorimetric sensor arrays for the efficient identification and sensitive detection of AGs.

Laccase is a multi-copper oxidase that can oxidize a wide range of phenolic and aromatic amine substrates without H_2_O_2_ production [36,37]. However, natural laccase is costly, challenging to extract and purify, and usually unstable. Researchers have been working on the development of laccase mimics to overcome these drawbacks [38,39]. In 2015, Ren et al. [40] reported on copper-containing carbon dots (Cu-CDs) with laccase-like activity for the first time, which can oxidize the phenolic compounds *p*-phenylenediamine and hydroquinone efficiently. Most of the currently reported nanomaterials with laccase-like activity are synthesized through inorganic, organic, or hybrid strategies by mimicking copper ions in the active center of natural laccase, such as Cu-doped Mn_3_O_4_ [41], I-Cu [42], Tris-Cu [43], GMP-Cu [44], and CH-Cu [45]. These materials show significant advantages in the catalytic degradation and detection of phenolic pollutants by mimicking the Cu-N/O coordination environment of natural laccase. Although laccase mimics have been widely studied in the field of catalysis, there are still few reports on non-copper-based laccase mimics, and their applications are mainly limited to the detection and degradation of phenolic compounds. It was found that other metal-based nanomaterials (Mn, Pt, and Ce) also exhibit laccase-like catalytic properties [46,47,48]. Notably, studies have shown that some silver-based nanomaterials also have laccase-like activity, such as silver citrate (AgCit) [49], Ag_2_O [50], and Ag_3_PO_4_ [51], which exhibit excellent performance in biosensing. Based on the enhancement in AgCit’s laccase-like activity, Zhou et al. [52] constructed a novel colorimetric aptasensor for the sensitive and highly selective detection of histamine. The aptasensor showed good linearity in the range of 20–600 μg·L^−1^ and has a limit of detection (LOD) as low as 27 μg·L^−1^. Therefore, it is of great research significance to design and construct new silver-based materials with laccase-like activity and explore their application value in the field of contaminant detection.

**Figure 1:**
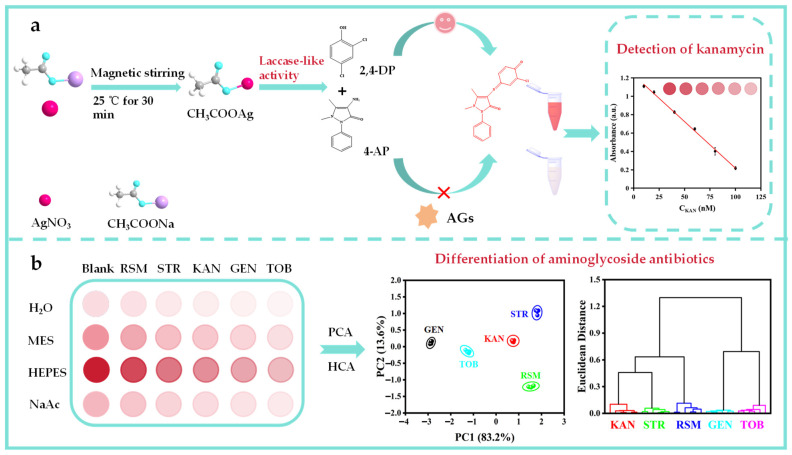
Experimental scheme. (**a**) Synthesis of CH_3_COOAg and its application in KAN detection. (**b**) Illustration of colorimetric sensor array for identifying various AGs.

In this work, a CH_3_COOAg material with superb laccase-like activity was prepared through a co-precipitation method (Figure 1a). The laccase-like activity of CH_3_COOAg was investigated using 2,4-dichlorophenol (2,4-DP) as the substrate and 4-aminoantipyrine (4-AP) as the color developer. It was found that the material exhibited different intensities of laccase-like activity in different solutions, and five AGs can inhibit the laccase-like activity of CH_3_COOAg to different degrees in different buffer systems. Therefore, a four-channel colorimetric sensor array was designed using four solutions as sensing channels for the efficient discrimination and sensitive detection of AGs (Figure 1b). The sensor array can accurately distinguish between different concentrations of the five AGs and their mixtures and perform well in the analysis of actual samples, such as tap and lake water. In addition, the laccase-like activity of CH_3_COOAg can also be used in the highly sensitive detection of KAN in a honey sample. Thus, the colorimetric sensor array constructed in this study has broad application prospects in the detection and identification of AGs.

## 2. Materials and Methods

### 2.1. Materials and Reagents

Details of the chemicals, reagents, and instrumentation used are provided in the Supplementary Materials, Text S1 and Text S2.

### 2.2. Preparation of CH_3_COOAg

CH_3_COOAg was synthesized through a direct precipitation method [53]. In brief, 10 mL of silver nitrate solution (1 M) was added to 10 mL of sodium acetate solution (1.2 M), and the mixture was stirred magnetically (425 rpm) for 30 min at 25 °C to produce a white precipitate. The product was then centrifuged at 4000 rpm for 10 min, and the precipitate was washed three times with deionized water. Finally, the precipitate was dried in a vacuum oven at 60 °C overnight, and the white product obtained was stored at room temperature (about 25 °C) protected from light.

### 2.3. An Investigation of the Laccase-like Activity of CH_3_COOAg

The laccase-like activity of the material was assessed using a colorimetric reaction between 2,4-DP and 4-AP. A total of 100 μL of CH_3_COOAg solution (2 mg/mL), 100 μL of 2,4-DP (1 mM), and 100 μL of 4-AP (1 mM) were added into 200 μL of deionized water, with a total volume of 500 μL. The mixed solution was placed in an oven and reacted at 70 °C for 10 min, and the absorbance of the solution at 510 nm was measured using a UV-Vis spectrophotometer. Then, the factors affecting the optimized reaction conditions of CH_3_COOAg were investigated using the control variable method, including the concentration of substrates 2,4-DP and 4-AP, the ratio of AgNO_3_ and CH_3_COONa for the synthesis of CH_3_COOAg, the type of buffer, the concentration of CH_3_COOAg, the pH value of the buffer, temperature, and the time of the reaction.

### 2.4. Steady-State Kinetic Analysis

The steady-state kinetics of CH_3_COOAg- and laccase-catalyzed 2,4-DP and 4-AP reactions were studied by varying the concentration of 2,4-DP and recording the absorbance value of the reaction solutions at 510 nm under the optimized experimental conditions. Typically, 100 μL of CH_3_COOAg (1.5 mg/mL), 100 μL of different concentrations of 2,4-DP (0.02–0.6 mM), and 100 μL of 4-AP (0.4 mM) were added to 200 μL of HEPES buffer (10 mM, pH = 7.0), with a total volume of 500 μL. After incubation at 25 °C for 10 min, the UV-Vis absorption spectra of the solutions were recorded, and the absorbance at 510 nm was measured. Catalytic kinetic experiments were also performed for natural laccase under optimized conditions (HEPES buffer (10 mM, pH 8.0), 70 °C for 10 min). The kinetic parameters (*K*_m_ and *V*_max_) were calculated using the Michaelis–Menten equation.

(1) $$V=\frac{V_{\max}\;\times \;[S]}{K_{m}+[S]}$$

Here, *V* is the initial reaction rate, *V*_max_ is the maximal reaction rate, [S] is the concentration of the substrate 2,4-DP, and *K*_m_ is the Michaelis–Menten constant [54].

### 2.5. Free Radical Scavenging Experiments

Free radical scavengers, such as isopropanol (IPA), L-histidine (L-His), and superoxide dismutase (SOD), were used to investigate the possible production of the reactive substances hydroxyl radical (•OH), singlet oxygen (^1^O_2_), and superoxide anion (O_2_^•−^) [55,56] from the CH_3_COOAg-catalyzed 2,4-DP and 4-AP systems, respectively. Different concentrations of each radical scavenger were added to 100 μL of CH_3_COOAg solution (1.5 mg/mL). Then, 100 μL of 2,4-DP (0.4 mM) and 100 μL of 4-AP solution (0.4 mM) were added to the mixed solution, and HEPES buffer (10 mM, pH = 7.0) was added to make a total volume of 500 μL. Finally, the reaction system was incubated at 25 °C for 10 min, and the absorbance value at 510 nm of the solution was measured.

### 2.6. Construction of CH_3_COOAg-Based Sensor Array for AG Recognition

Based on the different strengths of the laccase-like activity of CH_3_COOAg in H_2_O, MES, HEPES, and NaAc, a colorimetric array sensor was constructed using four sensing units (H_2_O, MES, HEPES, and NaAc) to identify five AGs (KAN, STR, TOB, GEN, and RSM). The procedure is as follows: 100 μL of CH_3_COOAg (1.5 mg/mL), 100 μL of 2,4-DP (0.4 mM), 100 μL of 4-AP (0.4 mM), 100 μL of HEPES/MES/NaAc/H_2_O (10 mM, pH = 7.0), and 100 μL of AGs (0.02–0.3 μM) were mixed (total volume of 500 μL) and incubated at 25 °C for 10 min. The experimental procedure for distinguishing between two-component AGs was the same as described above, except that single-component AGs were replaced by binary mixtures of different proportions. In addition, 100 μL of HEPES/MES/NaAc/H_2_O (10 mM, pH = 7.0), 100 μL of CH_3_COOAg (1.5 mg/mL), 100 μL of 2,4-DP (0.4 mM), 100 μL of 4-AP (0.4 mM), and 100 μL of real water samples spiked with AGs were mixed (the total volume should also be 500 μL). The UV-Vis absorption spectra at 700–400 nm of the reaction solutions were recorded. The absorbance value at 510 nm was measured and used in the differentiation of five AGs. The process was repeated six times each, and a training data matrix of 5 AGs × 4 channels × 6 repetitions was generated for each test. Principal component analysis (PCA) and cluster analysis (HCA) were conducted using Origin 2018 (Version: 9.5).

### 2.7. CH_3_COOAg-Based Colorimetric Sensor for Kanamycin (KAN) Detection

A colorimetric sensor based on CH_3_COOAg was developed for the detection of KAN in a honey sample. The honey sample was pretreated according to a study reported in the literature [57]. The specific operation is as follows: A total of 1.0 g of honey was dissolved in 5 mL of HEPES buffer (10 mM, pH = 7.0), and 4 mL of ethyl acetate was added. Ultrasonication was carried out for 15 min, after which the mixtures were centrifuged at 10,000 rpm for 10 min to remove the precipitates. The supernatant was then filtered through 0.22 μm filters three times. The solution was diluted 1000-fold with HEPES buffer and placed in a refrigerator at 4 °C before use. Different concentrations of KAN solutions were prepared in diluted honey sample solutions. Then, 100 μL of different concentrations of KAN solution was added to 100 μL of CH_3_COOAg (1.5 mg/mL), 100 μL of 2,4-DP (0.4 mM), 100 μL of 4-AP (0.4 mM), and 100 μL of HEPES buffer (10 mM, pH = 7.0). After incubation at 25 °C for 10 min, the UV-Vis absorption spectra of the solutions were recorded, and the absorbance at 510 nm was measured.

## 3. Results and Discussion

### 3.1. Characterization of CH_3_COOAg

**Figure 2:**
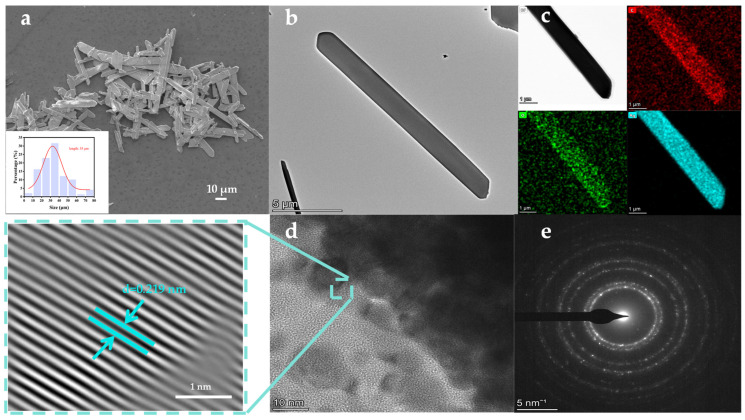
Surface morphology, element distribution, and microstructure of prepared materials. (**a**) SEM image (inset: particle size distribution), (**b**) TEM image, (**c**) EDS spectrum, (**d**) HRTEM image, and (**e**) SAED pattern of CH_3_COOAg.

Scanning electron microscopy (SEM) and transmission electron microscopy (TEM) were used to characterize the morphology of the prepared CH_3_COOAg material. The synthesized CH_3_COOAg is a columnar crystal with an average length of approximately 35 μm (Figure 2a) and a width of about 3.5 μm (Figure S1). The material appears to have a smooth surface (Figure 2b). High-resolution TEM (HRTEM) analysis reveals well-resolved lattice fringes with a lattice spacing of 0.219 nm (Figure 2d), and the corresponding selected area electron diffraction (SAED) patterns (Figure 2e) show a series of concentric electron diffraction rings, indicating that the columnar CH_3_COOAg is a crystalline material. The energy-dispersive X-ray spectroscopy (EDS) image (Figure 2c) and EDS results (Figure S2) of CH_3_COOAg reveal that elements C, O, and Ag are uniformly distributed in the material.

**Figure 3:**
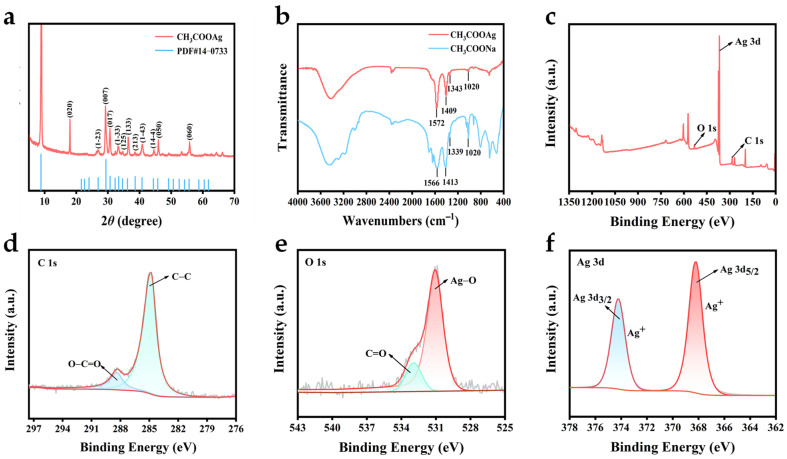
Structural characteristics of prepared materials. (**a**) XRD patterns of CH_3_COOAg. (**b**) FT-IR spectra of CH_3_COOAg and CH_3_COONa. (**c**) XPS survey spectra and high-resolution XPS spectra of CH_3_COOAg: (**d**) C 1s, (**e**) O1s, and (**f**) Ag 3d.

The crystal structure of CH_3_COOAg was investigated by X-ray diffraction (XRD). The diffraction peaks of CH_3_COOAg correspond to that of a standard card (PDF#14−0733) (Figure 3a). In reality, the previous literature reported that the unit cell for silver acetate is triclinic (space group P1, with unit cell dimensions of a = 5.5810; b = 9.960; c = 21.587; α = 89.10; β = 97.40; γ = 97.26) [58]. In addition, the individual diffraction peaks are sharp and well defined, indicating that the synthesized material is a crystalline structure, which supports the results of HRTEM analysis.

Fourier transform infrared spectroscopy (FT-IR) analysis was performed to further investigate the chemical bonding in CH_3_COONa and CH_3_COOAg (Figure 3b). In the FT-IR spectra of CH_3_COONa, the absorption peaks at 1020 and 1339 cm^−1^ are attributable to the rocking and in-plane bending (or deformation) of −CH_3_. The absorption peaks attributed to the symmetric and antisymmetric stretching of −COO− appear at 1413 and 1566 cm^−1^. Similar characteristic peaks are also found in the synthesized CH_3_COOAg with a slight shift in the peak position, indicating the successful coordination of silver ions (Ag^+^) and CH_3_COO^−^.

The elemental composition and atomic valence states of CH_3_COOAg were analyzed by XPS. The XPS full spectra (Figure 3c) show signals at 531.05, 367.62, and 284.32 eV for O 1s, Ag 3d, and C 1s, respectively, which indicate that the synthesized columnar CH_3_COOAg contains the three elements of O, Ag, and C, in agreement with the results of EDS analysis. The C, O, and Ag elements are present at about 11.2%, 43.0%, and 45.8%, respectively. In the high-resolution XPS spectra of C 1s (Figure 3d), the peaks at 284.8 and 288.38 eV indicate C−C and O−C=C, respectively. In the high-resolution XPS spectra of O 1s (Figure 3e), the characteristic peak with a binding energy of 531.0 eV indicates the presence of a Ag−O bond, and the peak corresponding to 532.93 eV indicates the presence of C=O in the synthesized material [50,59]. The high-resolution XPS spectra of Ag 3d are shown in Figure 3f; the peaks at 368.19 eV and 374.22 eV are ascribed to Ag 3d_5/2_ and Ag 3d_3/2_, respectively, revealing the presence of Ag^+^ in CH_3_COOAg. In addition, CH_3_COOAg was characterized thermogravimetrically to analyze its thermal decomposition process. The thermogravimetric analysis curve (Figure S3) shows that the mass loss of CH_3_COOAg is 36.31% at 252 °C, which is presumed to represent the oxidative decomposition of CH_3_COOAg upon heating, and the product is metallic silver [60]. The above characterization results indicate that the columnar crystal material CH_3_COOAg was successfully synthesized.

### 3.2. The Catalytic Activity of CH_3_COOAg and Laccase

**Figure 4:**
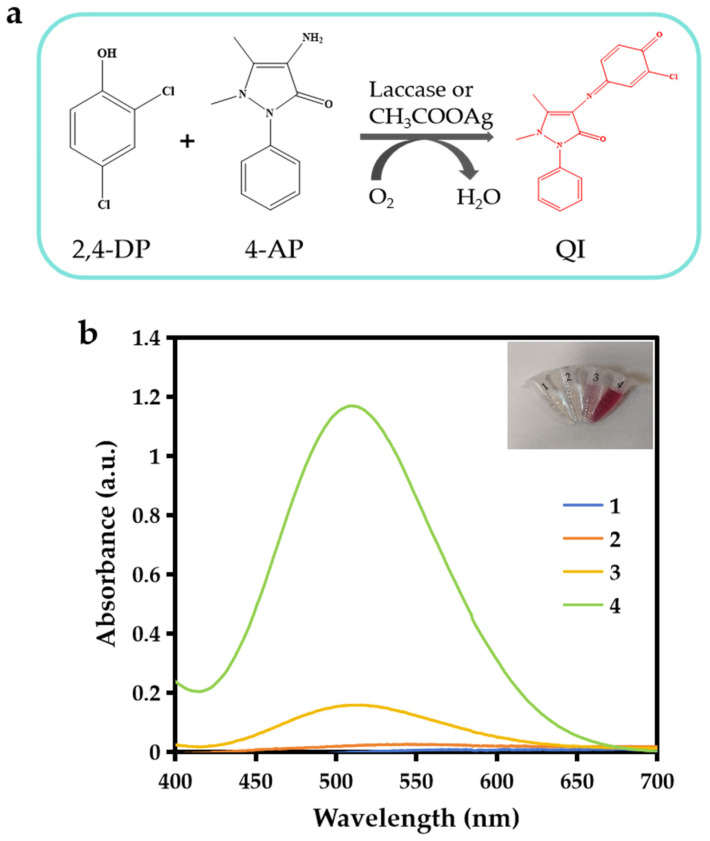
Laccase-like activity of prepared materials. (**a**) Schematic representation of reaction of laccase or CH_3_COOAg catalyzing oxidation of 2,4-DP with 4-AP. (**b**) Visual images and UV-Vis absorption spectra of reaction solutions (Sample 1: deionized water + 2,4-DP + 4-AP; Sample 2: Sample 1 + 2 mg·mL^−1^ CH_3_COONa; Sample 3: Sample 1 + 2 mg·mL^−1^ AgNO_3_; Sample 4: Sample 1 + 2 mg·mL^−1^ CH_3_COOAg).

The laccase-like activity of CH_3_COOAg was evaluated by the colorimetric reaction of the laccase substrate (2,4-DP) with chromogenic agent 4-AP, which produces red quinoneimine (QI) with an absorption peak at 510 nm (Figure 4a). As depicted in Figure 4b, when deionized water or CH_3_COONa was added to the 2,4-DP and 4-AP systems, the solutions were colorless with no absorption peak at 510 nm. However, after the addition of AgNO_3_ or CH_3_COOAg, a clear absorption peak at 510 nm was observed, and the solution underwent a significant color change. Specifically, the addition of CH_3_COOAg gave the solution a darker pink color and higher absorbance at 510 nm as compared to AgNO_3_, indicating that CH_3_COOAg has excellent laccase-like activity, and its catalytic activity stems from itself rather than the possible released free Ag^+^ or CH_3_COO^−^. The catalytic performance of the catalyst is influenced by a variety of factors. The experimental conditions were investigated to obtain the optimized catalytic activity of the catalyst. Firstly, the effect of 2,4-DP (substrate) and 4-AP (chromogenic agent) concentrations on the laccase-like activity of CH_3_COOAg was investigated (Figure S4a). When the same concentrations of 2,4-DP and 4-AP were increased simultaneously, the absorbance value of the solution at 510 nm was gradually increased, indicating an enhancement in the laccase-like activity of CH_3_COOAg. The concentration of 0.4 mM of 2,4-DP and 4-AP was used in the subsequent experiments to ensure the proper absorption at 510 nm of the reaction system. Furthermore, as shown in Figure S4b, CH_3_COOAg has the best laccase-like activity when the molar ratio of AgNO_3_ and CH_3_COONa was 1:1.2 for the synthesis of the material. In addition, the concentration of CH_3_COOAg is also an important factor that affects its laccase-like activity, as shown in Figure S4c; when the concentration of CH_3_COOAg was 1.5 mg/mL, it showed superior laccase-like activity.

The laccase-like activity of CH_3_COOAg in different buffers was investigated (Figure S4d). The laccase-like activity of the material in PBS and Tris-HCl buffers was lower than that in H_2_O, possibly because Ag^+^ can react with phosphate and chloride ions to produce silver phosphate and silver chloride, respectively. In contrast, the laccase-like activity of the material in HEPES, MES, and NaAc buffers was higher than that in H_2_O. It was found that the Zeta potentials of CH_3_COOAg in three buffers (HEPES, MES, and NaAc) were all lower than that in H_2_O (Figure S6). According to the literature [43], the pKa value is 7.51 for HEPES, 6.15 for MES, and 4.76 for NaAc. At pH = 7, H_2_O is electrically neutral, MES and NaAc are negatively charged, and HEPES is partially ionized. The enhanced catalytic activity of CH_3_COOAg in the three buffers may be because the electrostatic forces of HEPES, MES, and NaAc are conducive to the binding of CH_3_COOAg with 2,4-DP.

The effects of different pH values, temperatures, and incubation times on the catalytic activity of CH_3_COOAg and natural laccase were investigated. CH_3_COOAg had the best catalytic activity when the pH of the HEPES buffer was 7.0, and the optimized reaction pH for natural laccase was 8.0 (Figure S4e). To further investigate the effect of pH on CH_3_COOAg, the Zeta potential of CH_3_COOAg in the HEPES buffer with different pH values was investigated. The results show that the Zeta potential of CH_3_COOAg is −8.59 mV under the pH condition with optimum catalytic activity (Figure S7). Furthermore, the CH_3_COOAg prepared in this experiment exhibits good catalytic properties in a wide range of temperatures, and its laccase-like activity is better at low temperatures. As CH_3_COOAg was prepared under mild conditions, the subsequent experiments were conducted at a temperature of 25 °C. As shown in Figure S4f, the catalytic activity of natural laccase is better at high temperatures, and its optimized catalytic activity is seen at 70 °C. The absorbance at 510 nm of the CH_3_COOAg-catalyzed 2,4-DP and 4-AP reaction solutions is increased dramatically with increasing incubation time, and it remained essentially unchanged when the incubation time was greater than 10 min. In contrast, the absorbance at 510 nm of the natural laccase-catalyzed 2,4-DP and 4-AP systems is changed slowly with an increase in the incubation time, and the absorbance of the solution was only 0.1348 when the incubation time was 16 min (Figure S4g). Therefore, the prepared laccase mimic CH_3_COOAg has better catalytic activity than natural laccase. In summary, the optimized reaction conditions for CH_3_COOAg are as follows: 0.4 mM of 2,4-DP and 4-AP, a molar ratio of AgNO_3_ and CH_3_COONa of 1:1.2, 1.5 mg/mL of CH_3_COOAg, HEPES buffer (pH = 7.0), 25 °C, and a 10 min reaction time. In addition, the catalytic activity of CH_3_COOAg and laccase in ethanol was also investigated, and the results show that CH_3_COOAg was not as ethanol-tolerant as natural laccase (Figure S4h). The catalytic activity of the material is usually affected by different storage times, so the activity of CH_3_COOAg was examined for different storage times at room temperature. As shown in Figure S5, the catalytic activity of CH_3_COOAg remained above 85% after being stored at room temperature for 10 days, demonstrating excellent storage stability.

### 3.3. Catalytic Kinetics of CH_3_COOAg and Laccase

The kinetics of the enzymatic reaction catalyzed by CH_3_COOAg and natural laccase were investigated using 2,4-DP as the substrate. CH_3_COOAg and natural laccase can catalyze the color development of the 2,4-DP and 4-AP systems. By recording the absorbance at 510 nm with varying 2,4-DP concentrations, plots of absorbance versus 2,4-DP concentration were obtained (Figure S8a,c), and Lineweaver–Burk plots were derived using the double reciprocal expression (Figure S8b,d). According to the Lineweaver–Burk equation, *K*_m_ and *V*_max_ were calculated to be 0.030 mM and 9.36 μM/min for CH_3_COOAg and 0.091 mM and 3.25 μM/min for natural laccase, respectively. The CH_3_COOAg synthesized in this study has a lower *K*_m_ value, indicating a higher affinity of CH_3_COOAg to the substrate 2,4-DP, and a larger *V*_max_ value indicates a faster rate of the reaction catalyzed by CH_3_COOAg as compared to natural laccase and the reported laccase mimics (Table S1).

### 3.4. Catalytic Mechanism

To investigate the catalytic mechanism of the laccase-like activity of CH_3_COOAg, nitrogen gas was bubbled into the reaction solution to remove dissolved oxygen from the solution. As shown in Figure S9a, the absorbance of the nitrogen-bubbled solution at a wavelength of 510 nm was lower than that of the solution exposed to air, proving that oxygen is essential for the catalytic process of CH_3_COOAg.

The types of reactive oxygen species (ROS) produced in the reaction system were investigated through free radical trapping experiments. As shown in Figure S9b, the addition of IPA, L-His, and SOD to the reaction system can significantly inhibit its color development in a concentration-dependent manner. Therefore, the ROS present in the reaction may be hydroxyl radicals (•OH), single linear oxygen (^1^O_2_), and superoxide anion (O_2_^•−^). In addition, it is obvious that the catalytic activity of CH_3_COOAg is almost completely inhibited when only low concentrations of SOD were added to the reaction system. Thus, O_2_^•−^ plays a major role in the catalytic process of CH_3_COOAg.

Based on the above experimental results and the catalytic mechanism of laccase mimic AgMal reported by Wang et al. [61], a possible catalytic mechanism of CH_3_COOAg was hypothesized. As depicted in Figure S10, the process essentially consisted of three catalytic steps, including (1) substrate binding, (2) substrate oxidation, and (3) oxygen reduction. The substrate 2,4-DP is initially bound to CH_3_COOAg, where Ag^+^ accepts electrons provided by the substrate to be reduced to Ag^0^. Meanwhile, 2,4-DP is oxidized by Ag^+^ to semiquinone radicals, which then undergo electron rearrangement to become benzoquinone. Finally, the O_2_ in the catalytic system binds to Ag^0^, converting to H_2_O. At the same time, Ag^0^ is oxidized to Ag^+^, thereby completing the catalytic cycle of CH_3_COOAg.

### 3.5. Effect of AGs on Laccase-like Activity of CH_3_COOAg

To evaluate the feasibility of a CH_3_COOAg-based assay for AG differentiation, the effects of five common AGs, including KAN, STR, RSM, GEN, and TOB, on the laccase-like activity of CH_3_COOAg were investigated. As shown in Figure S11a, CH_3_COOAg exhibits different laccase-like activity levels in H_2_O, MES, NaAc, and HEPES. Moreover, the addition of five AGs had different inhibitory effects on the laccase-like activity of CH_3_COOAg in different solutions.

The interference study of the colorimetric sensor was conducted by the addition of substances commonly found in food that may interfere with the detection of AGs to the reaction system, including antibiotics (tetracycline, metronidazole, penicillin sodium, carbamazepine, and chloramphenicol), amino acids (L-lysine, L-phenylalanine, glycine, and L-serine), glucose, and ions (K^+^, Na^+^, Fe^3+^, Ca^2+^, Cd^2+^, Mg^2+^, and NO_3_^−^). As shown in Figure S11b, only the addition of AGs can inhibit the laccase-like activity of CH_3_COOAg to varying degrees, indicating the good selectivity of the colorimetric sensor for AG detection.

### 3.6. Mechanism of Inhibitory Effect of AGs on Laccase-like Activity of CH_3_COOAg

#### 3.6.1. Zeta Potential Analysis

The chemical structures of AGs are shown in Figure S12. AGs have the characteristics of polycations and are highly positively charged due to the presence of multiple amino groups in their structures under neutral pH conditions [62,63]. The inhibition mechanism of AGs in terms of the laccase-like activity of CH_3_COOAg was investigated using KAN as proof of concept. The Zeta potential change of CH_3_COOAg before and after the addition of KAN was determined. In the HEPES buffer solution (10 mM, pH = 7.0), the Zeta potential of CH_3_COOAg is about −8.59 mV, indicating that the prepared material is negatively charged under this condition. The Zeta potential of CH_3_COOAg increased to −5.18 mV after the addition of KAN (Figure S13a). The main reason for this phenomenon is that KAN can be adsorbed on the surface of CH_3_COOAg by electrostatic force, thereby reducing the negative charge density of CH_3_COOAg.

#### 3.6.2. Enzyme Kinetics

The effect of KAN on the catalytic kinetics of CH_3_COOAg was investigated by varying the substrate concentration. The double reciprocal plots of CH_3_COOAg were obtained through changing the concentrations of substrates and KAN. As shown in Figure S13b, when the concentration of 2,4-DP was changed, the intercept 1/*V*_max_ of the vertical axis gradually increased with an increase in the concentration of KAN, indicating that *V*_max_ gradually decreased. The absolute value of the intercept of the horizontal axis (1/*K*_m_) remains almost the same, indicating that *K*_m_ remains unchanged, which means that the addition of KAN did not affect the affinity between CH_3_COOAg and 2,4-DP. The steady-state kinetic parameters of CH_3_COOAg with different concentrations of KAN are summarized in Table S2. The *K*_m_ of the enzymatic reaction was almost unchanged before and after the addition of KAN, but *V*_max_ decreased obviously with an increase in KAN, which reveals the noncompetitive reversible inhibition of KAN on the laccase-mimic activity of CH_3_COOAg. This kinetic feature suggests that KAN may reduce the catalytic efficiency of CH_3_COOAg through binding to the enzyme–substrate (ES) complex or a free enzyme, rather than directly competing for the substrate binding site.

### 3.7. CH_3_COOAg-Based Sensor Array for AG Recognition

**Figure 5:**
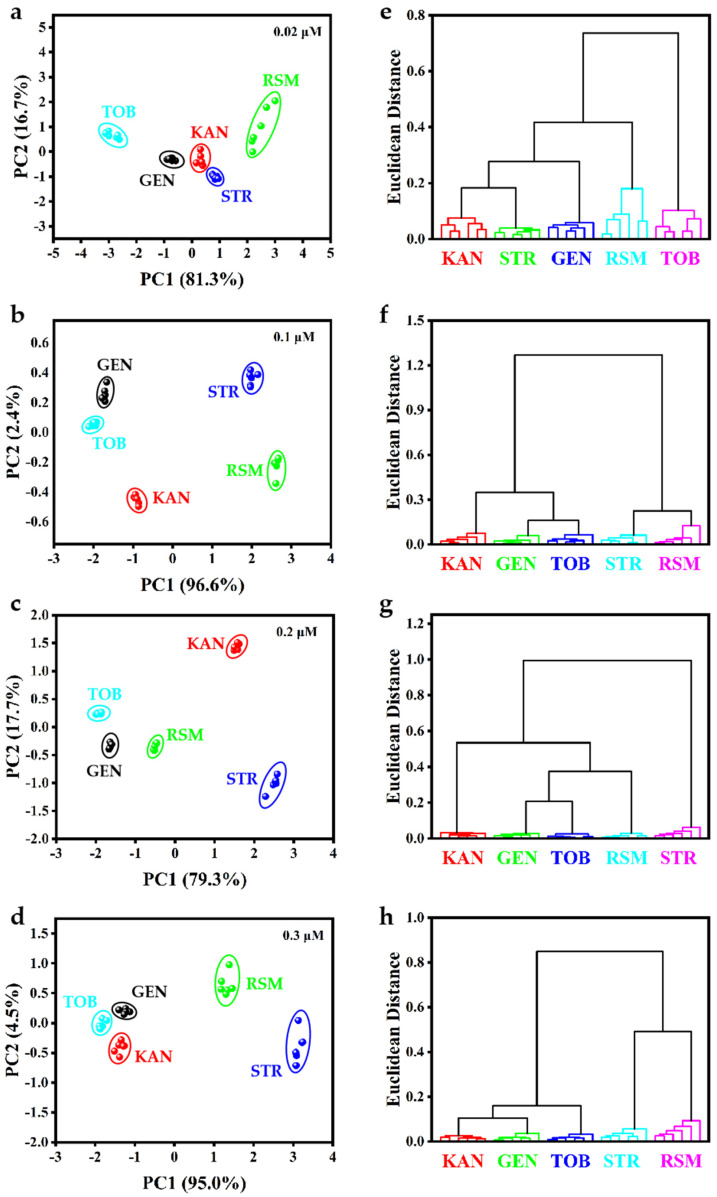
Distinguishing between single-component AGs. (**a**–**d**) Typical 2D PCA score plots and (**e**–**h**) HCA plots for five AGs at 0.02, 0.1, 0.2, and 0.3 µM, respectively.

Based on the different strengths of the laccase-like activity of CH_3_COOAg in different solutions and the fact that the addition of various AGs has different inhibitory effects on the laccase-like activity of CH_3_COOAg, a four-channel colorimetric sensor array was constructed using four solutions, including HEPES, MES, NaAc, and H_2_O, for the recognition of five AGs (KAN, STR, GEN, TOB, and RSM). The absorbance values at 510 nm for each channel were used as the output signal of the sensor array. The collected data matrix (4 signals × 5 AGs × 6 replicates) was subjected to PCA, and a two-dimensional (2D) scoring plot was obtained using the first two significant discriminators. As shown in Figure 5a–d, five AGs at different concentrations (0.02, 0.1, 0.2, and 0.3 μM) were clearly categorized into five distinct regions, and the confidence level is higher than 95%, indicating that the sensor array has excellent discrimination ability for five AGs. In addition, HCA was further applied to distinguish different species of AGs. As shown in Figure 5e–h, each AG at the same level was well categorized with no cross-talk between different AGs. The results indicate that the sensor array constructed based on four solutions can successfully discriminate various AGs at various concentrations.

Multiple antibiotics often coexist in real samples, so the identification of mixtures of AGs is an important indicator for evaluating the differential ability of the sensor array. To evaluate the ability for binary mixture identification, different molar ratios of GEN and STR were investigated. As shown in Figure S14, mixtures of GEN and STR (GEN/STR = 25/75, 50/50, and 75/25, with a total concentration of 0.1 μM), as well as 0.1 μM of GEN and STR, can be differentiated. In addition, binary mixtures of GEN and RSM with different molar ratios (GEN/RSM = 25/75, 50/50, and 75/25, with a total concentration of 0.1 μM), as well as 0.1 μM of GEN and RSM, can also be identified. The results show that the constructed sensor array can be used not only for the differentiation of single-component AGs but also for the identification of mixtures of binary AGs, and it may be further used in complex environment samples.

**Figure 6:**
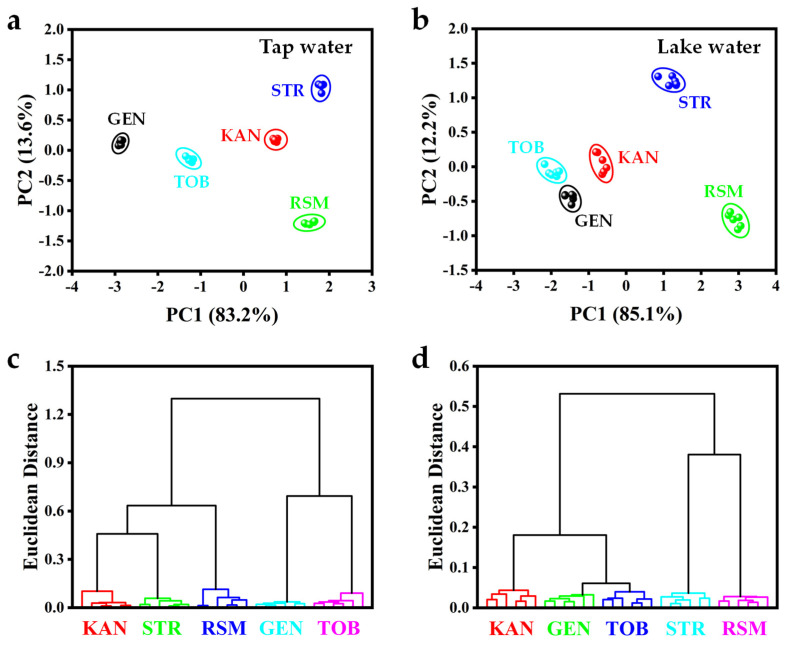
Distinguishing five AGs in actual water samples. (**a**) A 2D PCA score plot and (**c**) HCA plot for five AGs (1 µM) in tap water. (**b**) A 2D PCA score plot and (**d**) HCA plot for five AGs (0.4 µM) in lake water.

The feasibility of the sensor array to distinguish AGs in real samples was verified. The concentrations of AGs in tap water and lake water are 1 μM and 0.4 μM, respectively. Colorimetric assays for various AG components in tap and lake water were conducted, and the obtained colorimetric response patterns were converted to 2D scoring plots by PCA. As shown in Figure 6a,b, five AG components in tap and lake water were successfully categorized into five distinct regions. The HCA diagram also shows that tap and lake water containing different AGs can be divided into their respective groups (Figure 6c,d). Therefore, the designed sensor array can distinguish AGs in real water samples and has good application prospects.

### 3.8. Detection of KAN

**Figure 7:**
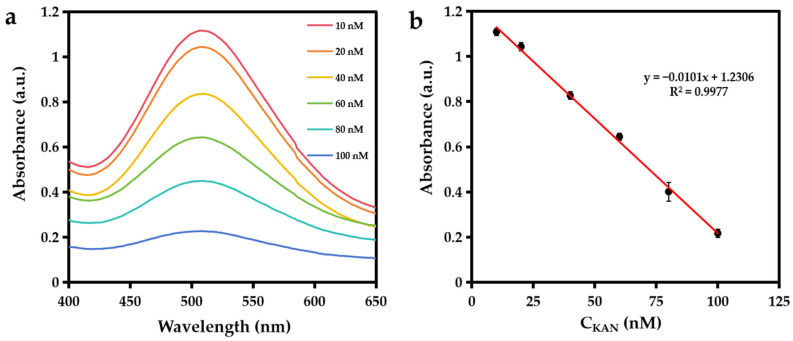
The detection of KAN. (**a**) The UV-Vis spectra of the CH_3_COOAg solutions in the presence of 10–100 nM of KAN in HEPES buffer (10 mM, pH = 7.0). (**b**) The relationship between KAN concentration and the corresponding absorption intensity at 510 nm.

The standard addition method was used to detect KAN in honey. As shown in Figure 7a, the absorbance at 510 nm of the solution decreased gradually with an increase in the concentration of added KAN, and the absorbance value shows a good linear relationship with the concentration of KAN in the range of 10–100 nM (*y* = −0.0101*x* + 1.2306; *R*^2^ = 0.9977). The detection limit of KAN is 3.99 nM (Figure 7b). As summarized in Table 1, the developed colorimetric method has a lower LOD than that of previously reported KAN sensing methods, highlighting its superior sensitivity. This excellent performance is primarily attributed to the exceptional laccase-mimicking activity of CH_3_COOAg at low concentrations. KAN suppresses the CH_3_COOAg-catalyzed oxidation of 2,4-DP, diminishing the reaction system’s signal. By leveraging this enzyme inhibition mechanism, the sensor achieves amplified responsiveness to trace KAN, resulting in highly sensitive detection. Moreover, the colorimetric assay was applied in the spiked recovery test of a honey sample, and the recoveries are 91.5–94.4% with an RSD < 6.8% (Table 2), indicating that the developed colorimetric detection method can be used for the detection of KAN in real samples.

## 4. Conclusions

In summary, a novel silver-based CH_3_COOAg material with laccase-like activity was successfully synthesized through a simple precipitation reaction using silver nitrate and sodium acetate as precursors. Compared with natural laccase and other reported laccase mimics, CH_3_COOAg has lower *K*_m_ and higher *V*_max_ values, indicating that CH_3_COOAg has excellent catalytic performance. A four-channel colorimetric sensor array was constructed using four solutions (H_2_O, HEPES, MES, and NaAc) as the sensing units, which can successfully differentiate between single-component (0.02–0.3 μM) and two-component mixtures of AGs, as well as distinguishing the five AGs in real water samples. In addition, a colorimetric method was established for the detection of KAN in a honey sample using the standard addition method, which has a linear range of 10–100 nM and an LOD of 3.99 nM. In short, this study not only extends the methods for the colorimetric detection and differentiation of AGs, but it also provides a reference for the design and development of more non-copper materials with laccase-like activity in the future.

## Figures and Tables

**Table 1:** Comparison of different KAN detection methods.

Method	Material System	Linear Range (μM)	LOD (nM)	Ref.
Colorimetry	CH_3_COOAg *	0.01–0.1	3.99	This work
Colorimetry	CuNCs@SiO_2_-DNA-AgNCs *	0.1–0.8	14.5	[64]
Colorimetry	WS_2_ Nanosheets *	0.1–0.5	60	[65]
Colorimetry	AuNPs *	1–40	680	[66]
Fluorescence	Y-CDs *	6.66–66.6	51.9	[67]
Fluorescence	AuNPs *	0.2–10	71.53	[68]
Fluorescence	NPG/MCH/Cat-Chit *	0.1–0.8	23.6	[69]
Fluorescence	CDs *	0.04–0.24	18	[70]
Electrochemistry	Co/Ni-Bio-MOF-ZrO_2_@Au *	10–1000	37	[71]
Electrochemistry	UiO-66-NH_2_/MCA/MWCNT@rGONR *	0.025–0.9	13	[72]
Surface Plasmon Resonance	rGO *	1–100	285	[73]

* CH_3_COOAg: Silver acetate; CuNCs@SiO_2_-DNA-AgNCs: Silica nanoparticles (SiO_2_) combined with copper nanoclusters (CuNCs) and DNA-templated silver nanoclusters (DNA-AgNCs); WS_2_ Nanosheets: Tungsten disulfide nanosheets; AuNPs: Gold nanoparticles; Y-CDs: Yellow-emitting carbon dots; NPG/MCH/Cat-Chit: Catechol–chitosan redox capacitor constructed on MCH SAM-modified nanoporous gold; CDs: Carbon dots; Co/Ni-Bio-MOF-ZrO_2_@Au: Cobalt–nickel bio-metal–organic framework and gold-doped zirconium dioxide; UiO-66-NH_2_/MCA/MWCNT@rGONR: Nanocomposite comprising an amine-functionalized metal–organic framework (UiO-66-NH_2_), a multiwalled carbon nanotube@reduced graphene oxide nanoribbon (MWCNT@rGONR), and a covalent organic framework (COF) synthesized using melamine and cyanuric acid monomers via polycondensation (represented by MCA); rGO: Reduced graphene oxide.

**Table 2:** Detection of kanamycin in honey sample.

Added (nM)	Found (nM)	Recovery (%)	RSD (%) (*n* = 3)
0	-	-	-
20	18.8	94.0	6.8
40	36.6	91.5	2.4
60	56.6	94.4	2.6

## Data Availability

No new data were created.

## Supplementary Materials

The following supporting information can be downloaded at: https://www.mdpi.com/article/10.3390/bios15090570/s1, Text S1: Chemicals and reagents; Text S2: Instrumentation; Table S1: Comparison of kinetic parameters of various laccase mimics; Table S2: Kinetic parameters of CH_3_COOAg with different concentrations of KAN; Figure S1: Width distribution diagram of CH_3_COOAg; Figure S2: EDS result of CH_3_COOAg; Figure S3: Thermogravimetric analysis curve of CH_3_COOAg; Figure S4: Catalytic activity of CH_3_COOAg and laccase. Effect of different (a) concentrations of 2,4-DP and 4-AP, (b) feeding ratios of AgNO_3_ to CH_3_COONa, (c) CH_3_COOAg concentrations, and (d) buffers on laccase-like activity of CH_3_COOAg. Catalytic activity of CH_3_COOAg and laccase at different (e) pHs, (f) temperatures, (g) reaction times, and (h) ethanol concentrations; Figure S5: Relative activity of CH_3_COOAg at different storage times. Figure S6: Zeta potential of CH_3_COOAg in different buffers (10 mM, pH = 7.0) and H_2_O; Figure S7: Zeta potential of CH_3_COOAg in 10 mM of HEPES buffer at different pHs (adjusting pH with 1 M HCl and 1 M NaOH); Figure S8: Catalytic kinetics of prepared material and laccase. Relationship between 2,4-DP concentration and corresponding absorption intensity at 510 nm: (a) CH_3_COOAg and (c) laccase. Corresponding linear Lineweaver–Burk plot of (b) CH_3_COOAg and (d) laccase; Figure S9: (a) UV-Vis absorption spectra of CH_3_COOAg-catalyzed laccase reaction under air and nitrogen. (b) Effects of various free radical scavengers on catalysis of 2,4-DP by CH_3_COOAg; Figure S10: Possible catalytic mechanism of CH_3_COOAg; Figure S11: (a) Effect of AGs on laccase-like activity of CH_3_COOAg in different solutions. (b) Impacts of various interfering substances (1 μM) on laccase-like activity of CH_3_COOAg; Figure S12: Chemical structures of five AGs; Figure S13: Inhibition mechanism of KAN on CH_3_COOAg. (a) Lineweaver–Burk plot for oxidation of 2,4-DP catalyzed by CH_3_COOAg in presence of KAN. (b) Zeta potential of CH_3_COOAg before and after addition of KAN. Figure S14: (a: GEN and STR; b: GEN and RSM) PCA diagram for discrimination of two-component AGs; (c: GEN and STR; d: GEN and RSM) HCA diagram for discrimination of two-component AGs. References [56,74,75,76,77,78,79,80,81,82] are cited in the Supplementary Materials.
