# Supplementary material for: CH3COOAg with Laccase-like Activity for Differentiation and Detection of Aminoglycoside Antibiotics
Source: Biosensors (Basel). 2025 Sep 1;15(9):570. doi: 10.3390/bios15090570 (PMC12467300; doi:10.3390/bios15090570)
Supplement: Supplementary file 1 [file biosensors-15-00570-s001.zip › biosensors-3794343-supplementary.pdf]

Supplementary Materials

CH_3COOAg with laccase-like activity for differentiation and detection of aminoglycoside antibiotics

Huan Zhu [a], Tong-Qing Chai [a], Jia-Xin Li [a], Jing-Jing Dai [a], Lei Xu [a], Wen-Ling Qin [b,**], Feng-Qing Yang [a,*]

[A] School of Chemistry and Chemical Engineering, Chongqing University, Chongqing 401331, China
[B] Chongqing Key Laboratory of Natural Product Synthesis and Drug Research, School of Pharmaceutical Sciences, Chongqing University, Chongqing 401331, China
* Corresponding Authors: Prof. Dr. Feng-Qing Yang, School of Chemistry and Chemical Engineering, Chongqing University, Chongqing 401331, China.

E-mail: fengqingyang@cqu.edu.cn.

Prof. Dr. Wen-Ling Qin, School of Pharmaceutical Sciences, Chongqing University.

E-mail: wenling.qin@cqu.edu.cn.

Supplementary Texts

Text S1. Chemicals and reagents.

Text S2. Instrumentation.

Supplementary Tables

Table S1. Comparison of kinetic parameters of various laccase mimics.

Table S2. Kinetic parameters of CH_3COOAg with different concentrations of KAN.

Supplementary Figures

Figure S1. Width distribution diagram of CH_3COOAg.

Figure S2. EDS results of CH_3COOAg.

Figure S3. Thermogravimetric analysis curve of CH_3COOAg.

Figure S4. Catalytic activity of CH_3COOAg and laccase. Effect of different (a) concentrations of 2,4-DP and 4-AP, (b) feeding ratios of $AgNO_3$ to CH_3COONa, (c) CH_3COOAg concentrations, and (d) buffers on laccase-like activity of CH_3COOAg. Catalytic activity of CH_3COOAg and laccase at different (e) pHs, (f) temperatures, (g) reaction times, and (h) ethanol concentrations.

Figure S5. Relative activity of CH_3COOAg at different storage times.

Figure S6. Zeta potential of CH_3COOAg in different buffers (10 mM, pH = 7.0) and H_2O.

Figure S7. Zeta potential of CH_3COOAg in 10 mM of HEPES buffer at different pHs (adjusting pH with 1 M HCl and 1 M NaOH).

Figure S8. Catalytic kinetics of prepared material and laccase. Relationship between 2,4-DP concentration and corresponding absorption intensity at 510 nm: (a) CH_3COOAg and (c) laccase. Corresponding linear Lineweaver–Burk plot of (b) CH_3COOAg and (d) laccase.

Figure S9. (a) UV-Vis absorption spectra of CH_3COOAg-catalyzed laccase reaction under air and nitrogen. (b) Effects of various free radical scavengers on catalysis of 2,4-DP by CH_3COOAg.

Figure S10. Possible catalytic mechanism of CH_3COOAg.

Figure S11. (a) Effect of AGs on laccase-like activity of CH_3COOAg in different solutions. (b) Impacts of various interfering substances (1 μM) on laccase-like activity of CH_3COOAg.

Figure S12. Chemical structures of five AGs.

Figure S13. Inhibition mechanism of KAN on CH_3COOAg. (a) Lineweaver–Burk plot for oxidation of 2,4-DP catalyzed by CH_3COOAg in presence of KAN. (b) Zeta potential of CH_3COOAg before and after addition of KAN.

Figure S14. (a: GEN and STR; b: GEN and RSM) PCA diagram for discrimination of two-component AGs; (c: GEN and STR; d: GEN and RSM) HCA diagram for discrimination of two-component AGs.

Supplementary Texts

Text S1. Chemicals and reagents.

Sodium acetate anhydrous (NaAc, AR) and isopropanol (IPA, AR) were purchased from Chongqing Chuandong Chemical (Group) Co., Ltd. Silver nitrate ($AgNO_3$, GR), ethyl alcohol (EtOH, AR), 1 M hydrochloric acid (1 MHCl, AR), sodium chloride (NaCl, ≥99.5%), potassium chloride (KCl, ≥99.5%), 10% phosphoric acid (10% H_3PO_4, AR), and magnesium sulfate heptahydrate ($MgSO_4·7H_2O$, ≥99.5%) were purchased from Chengdu Chron Chemicals Co., Ltd. Laccase (120 U/g, AR), superoxide dismutase (SOD, BR, 6000 u/mg), sodium penicillin (USP grade, 1603 U/mg), and D(+)-glucose (Glu, 99%) were purchased from Shanghai Yuan Ye Biological Technology Co., Ltd. 2,4-dichioropherol (2,4-DP, 98%), 2-morpholinoethanesulfonic acid (MES, 98%), and N-(2-hydroxyethyl) piperazine-N'-2-ethanesulfonic acid (HEPES, 99.5%) were purchased from Meryer Chemical Technology Co., Ltd. 4-aminoantipyine (4-AP, 98.5%), ferric chloride ($FeCl_3$, 99%), and chloromycetin (98%) were purchased from Beijing Mreda Technology Co., Ltd. Kanamycin (KAN, 98%) and ribostamycin (RSM, 98+%) were purchased from Shanghai Yien Chemical Technology Co., Ltd. Tobramycin (TOB, 98%), streptomycin (STR, 90%), tetracycline (TC, CP (Chinese Pharmacopoeia)), Flagyl (MNZ, 99%), and cadmium chloride ($CdCl_2·5/2H_2O$, 98%) were purchased from Shanghai Macklin Biochemical Co., Ltd. Gentamicine (GEN ≥590 IU/mg) was purchased from Solarbio. Sodium dihydrogen phosphate (NaH_2PO_4, ≥99.0%) and 1 M sodium hydroxide (1 M NaOH, ≥98.0%) were purchased from Shanghai Titan Technology Co., Ltd. Tris (hydroxymethyl) methyl aminomethane (Tris, AR, 99.9%) was purchased from BBI Life Sciences Co., Ltd. L-Histidine (L-His, 99%) and L-lysine (L-Lys, BR, 98%) were purchased from Chengdu Huaxia Chemical Reagent Co., Ltd. L-Phenylalanine (L-Phe, 99.5%) was purchased from Shanghai Sangon Biotech Co., Ltd. Glycine (Gly, ≥99%) was purchased from Shanghai Diebold Biotechnology Co., Ltd. L-serine (L-Ser, 99%) was purchased from Tianjin Xien Si Biochemical Technology Co., Ltd. Carbamazepine (＞98%) was purchased from Dalian Meilun Biotechnology Co., Ltd. Calcium chloride ($CaCl_2$, ≥96.0%) was purchased from Tianjin Damao Chemical Reagent Factory. All chemicals were used as received without further purification. The honey sample was purchased from Guizhou Qianmiyun Zhongfeng Honey Industry Technology Co., Ltd. The ultrapure water (18.25 MΩ·cm) used for all the experiments was prepared using a water purification system (ATSelem 1820A, Antesheng Environmental Protection Equipment Co., Ltd.). The actual tap water sample was from our laboratory, and lake water was from Yun Lake of Chongqing University.

Text S2. Instrumentation.

Scanning electron microscopy (SEM) images were obtained using a field-emission scanning electron microscope (FESEM) (Quanta 650, FEI, Hillsboro, OR). Transmission electron microscopy (TEM) images and element distribution analysis results were recorded using a JEM 2100 electron microscope (JEOL Ltd. Tokyo, Japan) working at 200 kV, which is equipped with an energy-dispersive X-ray spectrometer (EDX). X-ray diffraction (XRD) patterns were obtained using an X'pert Powder diffractometer (Malvern Panalytical Ltd., Netherlands) with secondary beam graphite monochromated Cu Kα radiation. X-ray photoelectron spectrometry (XPS) was recorded on a PHI5000 Versaprobe system using monochromatic Al Ka radiation (1486.6 eV), and the obtained binding energies were referenced to the C 1s line set at 284.8 eV (Thermo Fisher Scientific Ltd, UK). Fourier transform infrared spectra (FT-IR) were taken on a Nicolet iS50 spectrometer (Thermo Fisher Scientific, USA) between 4000 cm^{-1} and 400 cm^{-1} in KBr media. A 0.22 μm microporous nylon membrane was purchased from Shanghai Titan Technology Co., Ltd. (Shanghai, China). An ultrasonic cleaner (KS-3200B) was purchased from Kunshan Jielimei Ultrasonic Instrument Co., Ltd. (Hangzhou, China). A vacuum drying oven (DZF−6012) was purchased from Shanghai Yiheng Scientific Instruments Co., Ltd. (Shanghai, China). An analytical balance (ATX124) was purchased from Shimadzu (Japan). A UV-Vis spectrophotometer (UV-5500) was obtained from Shanghai Metash Instruments Co., Ltd. (Shanghai, China). A magnetic stirrer (ZGCJ−3A) was purchased from Shanghai Zigui Instruments Co., Ltd. (Shanghai, China). A benchtop low-speed centrifuge (L420) was purchased from Hunan Xiangyi Laboratory Instrument Development Co., Ltd. (Hunan, China).

Supplementary Tables

Table S1. Comparison of kinetic parameters of various laccase mimics.

Materials	K_m (mM)	V_{max} (μM/min)	Ref.
GSH-Cu	6.37	2.30	[74]
ATP-Cu	0.207	2.2	[75]
CA-Cu	0.12	7.82	[76]
Bpy-Cu	0.19	1.48	[77]
Cu-Mn MOF	0.080	1.315	[78]
Cu-BH	0.09	7.81	[56]
Cu-Cys NLs	0.14	1.44	[79]
Cu_2O	0.2	6.5	[80]
CuNAD	0.068	6.28	[81]
MBI-Cu	0.107	5.80	[82]
CH_3COOAg	0.030	9.36	This work
Laccase	0.091	3.25	This work

GSH-Cu: Complex of glutathione with copper; ATP-Cu: Complex of adenosine triphosphate with copper; CA-Cu: Complex of cysteine–aspartic dipeptide with copper; BPy-Cu: Complex of 4,4′-bipyridine with copper; Cu-Mn MOF: Copper–manganese metal–organic framework; Cu-BH: Complex of 2-aminoterephthalic acid and histidine with copper; Cu-Cys NLs: Complex (nanoleaves) of cysteine with copper; Cu_2O: Cuprous oxide; CuNAD: Complex of nicotinamide adenine dinucleotide with copper; MBI-Cu: Complex of 2-methylbenzimidazole with copper.

Table S2. Kinetic parameters of CH_3COOAg with different concentrations of KAN.

KAN (μM)	Substrate	K_m (mM)	V_{max} (μM/min)
0		0.030	9.36
0.05	2,4-DP	0.027	6.75
0.1		0.020	3.50

Supplementary Figures

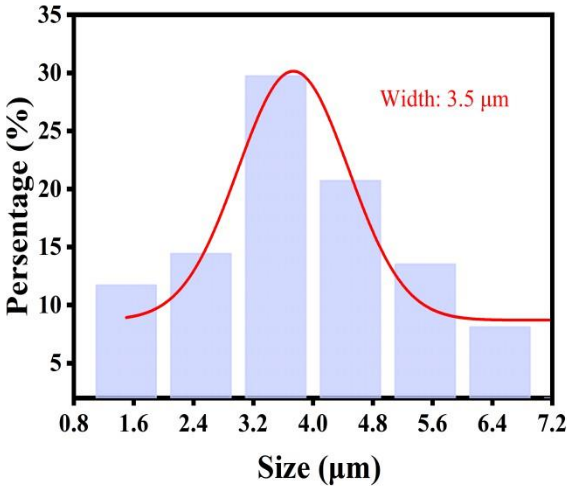

Figure S1. Width distribution diagram of CH_3COOAg.

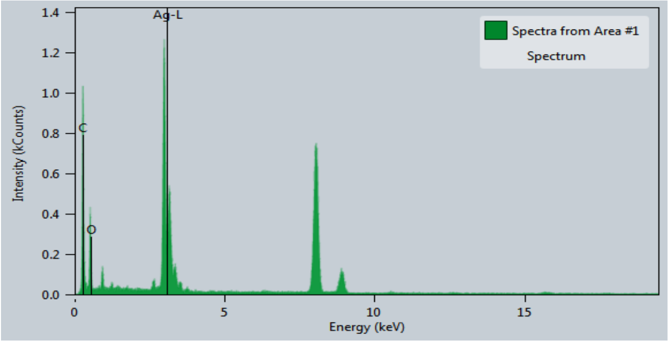

Figure S2. EDS results of CH_3COOAg.

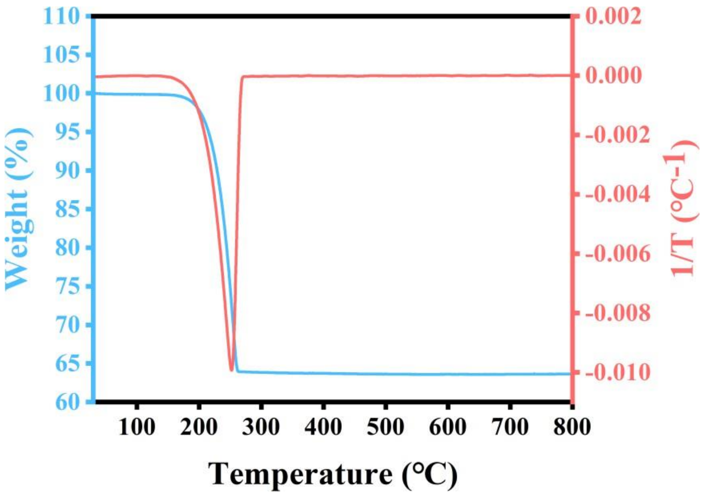

Figure S3. Thermogravimetric analysis curve of CH_3COOAg.

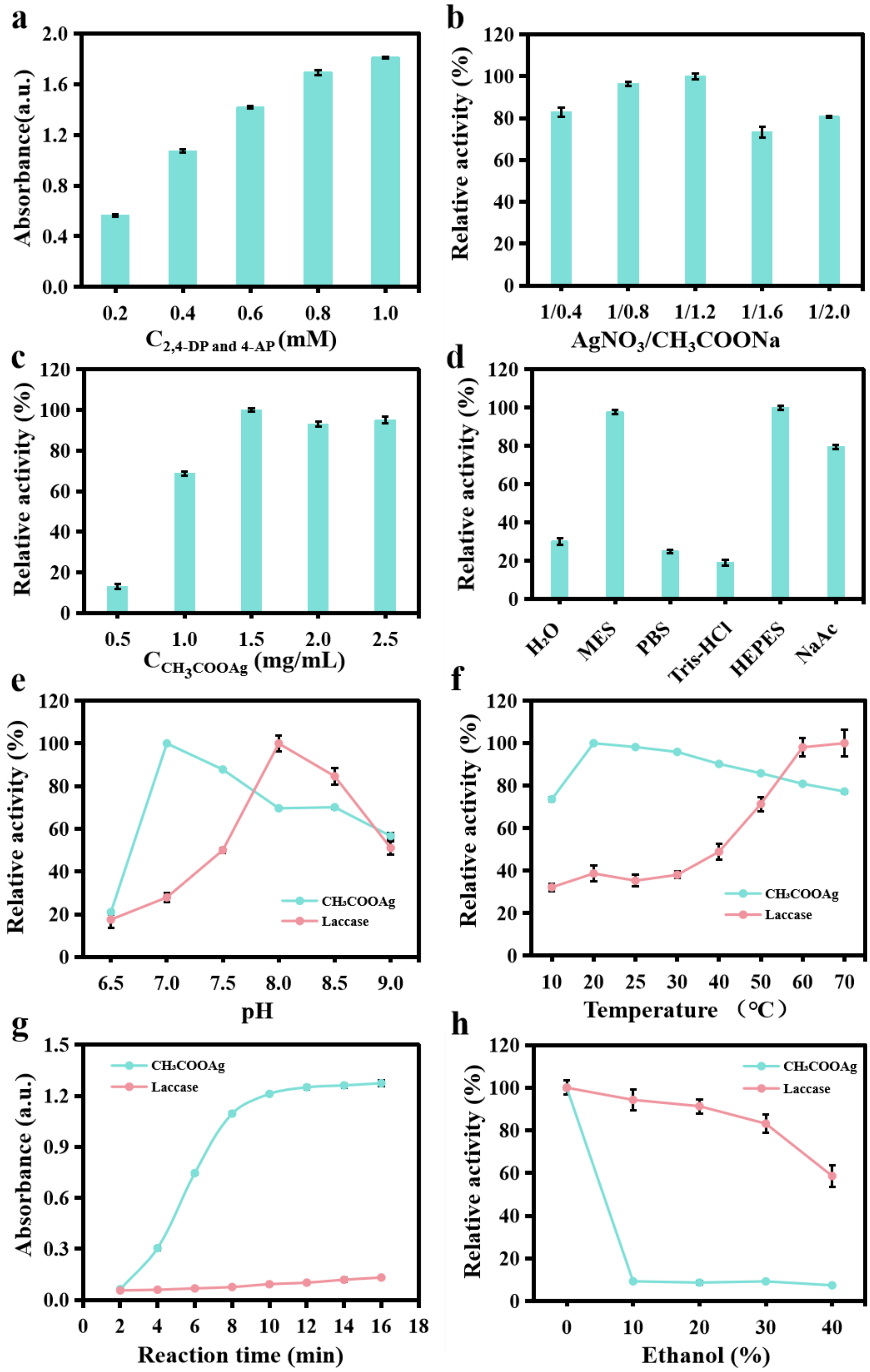

Figure S4. Catalytic activity of CH_3COOAg and laccase. Effect of different (a) concentrations of 2,4-DP and 4-AP, (b) feeding ratios of $AgNO_3$ to CH_3COONa, (c) CH_3COOAg concentrations, and (d) buffers on laccase-like activity of CH_3COOAg. Catalytic activity of CH_3COOAg and laccase at different (e) pHs, (f) temperatures, (g) reaction times, and (h) ethanol concentrations.

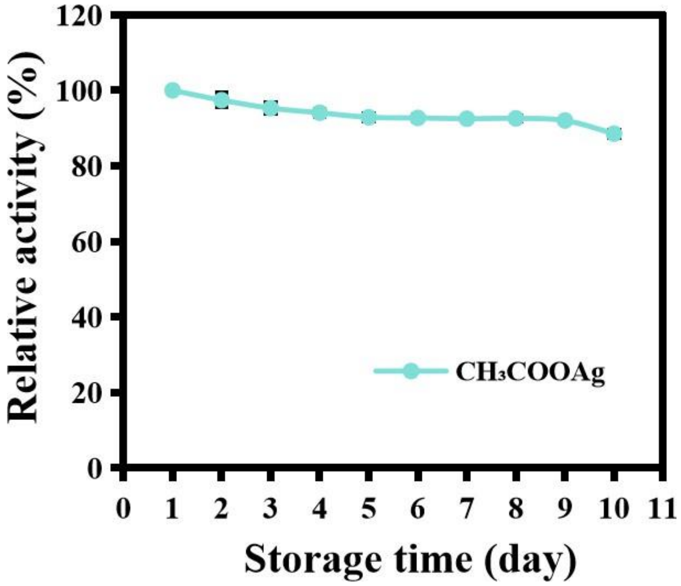

Figure S5. Relative activity of CH_3COOAg at different storage times.

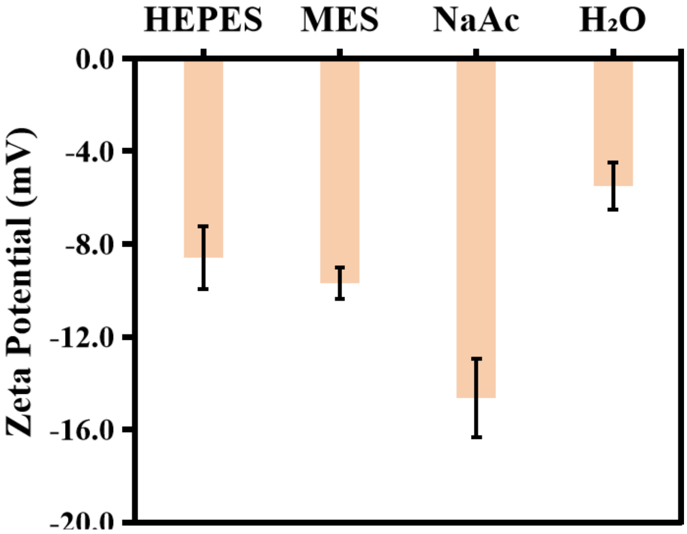

Figure S6. Zeta potential of CH_3COOAg in different buffers (10 mM, pH = 7.0) and H_2O.

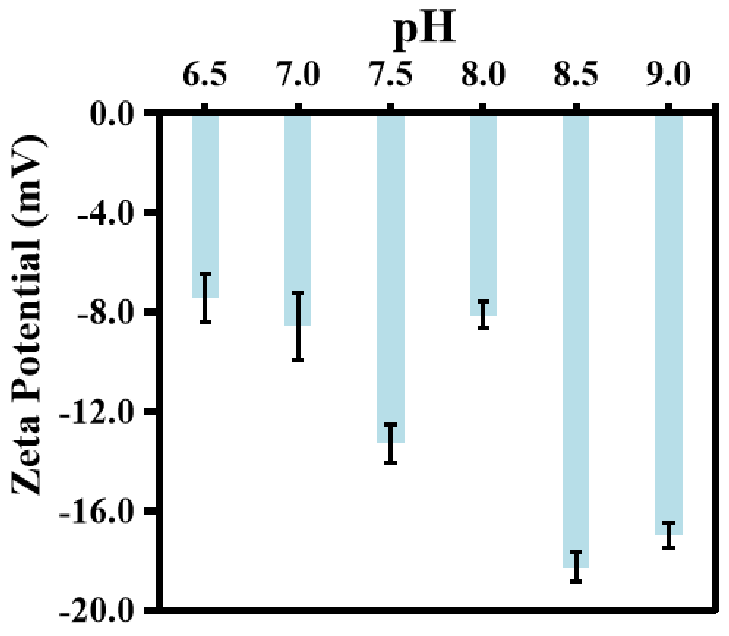

Figure S7. Zeta potential of CH3COOAg in 10 mM of HEPES buffer at different pHs (adjusting pH with 1 M HCl and 1 M NaOH).

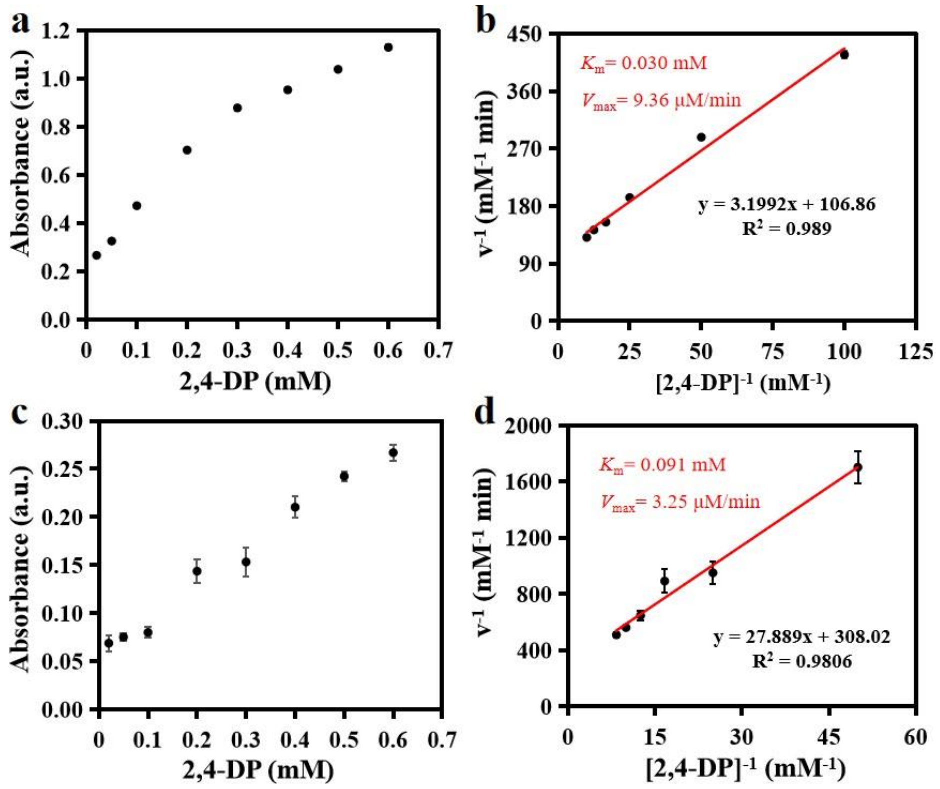

Figure S8. Catalytic kinetics of prepared material and laccase. Relationship between 2,4-DP concentration and corresponding absorption intensity at 510 nm: (a) CH_3COOAg and (c) laccase. Corresponding linear Lineweaver–Burk plot of (b) CH_3COOAg and (d) laccase.

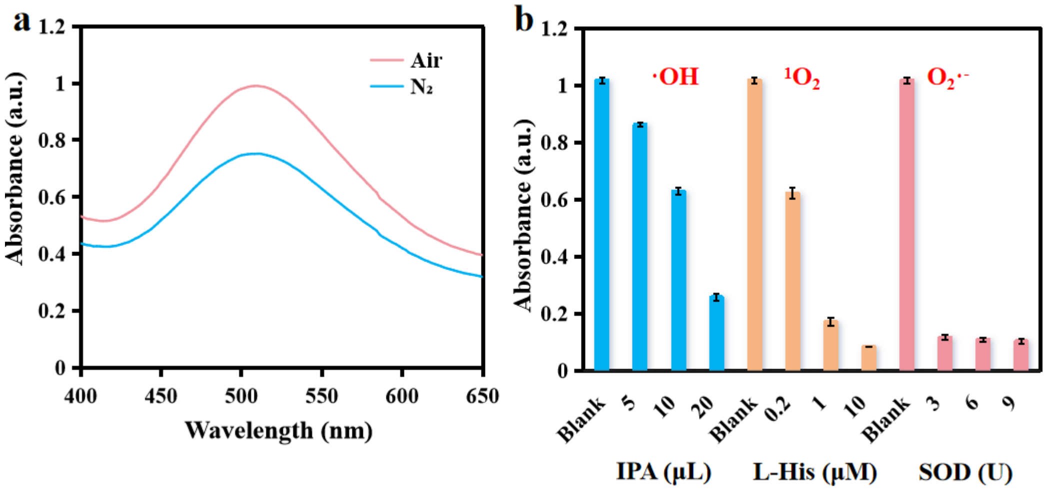

Figure S9. (a) UV-Vis absorption spectra of CH_3COOAg-catalyzed laccase reaction under air and nitrogen. (b) Effects of various free radical scavengers on catalysis of 2,4-DP by CH_3COOAg.

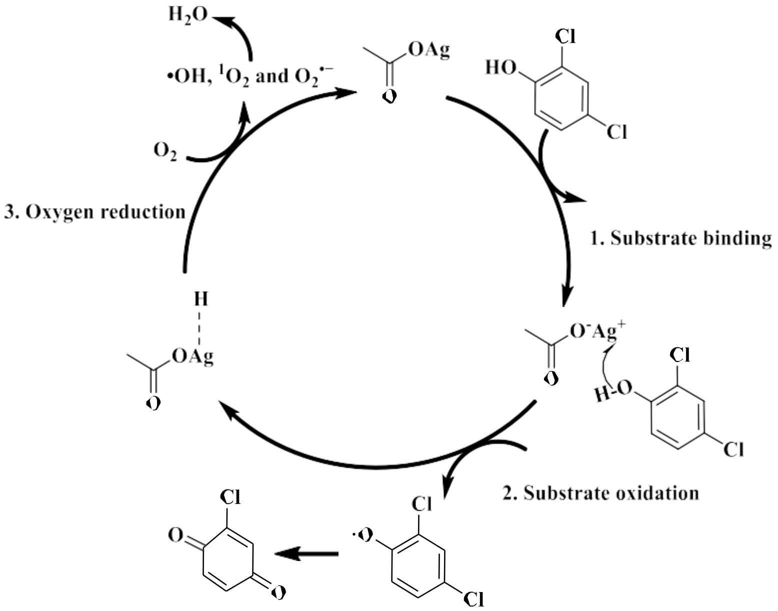

Figure S10. Possible catalytic mechanism of CH_3COOAg.

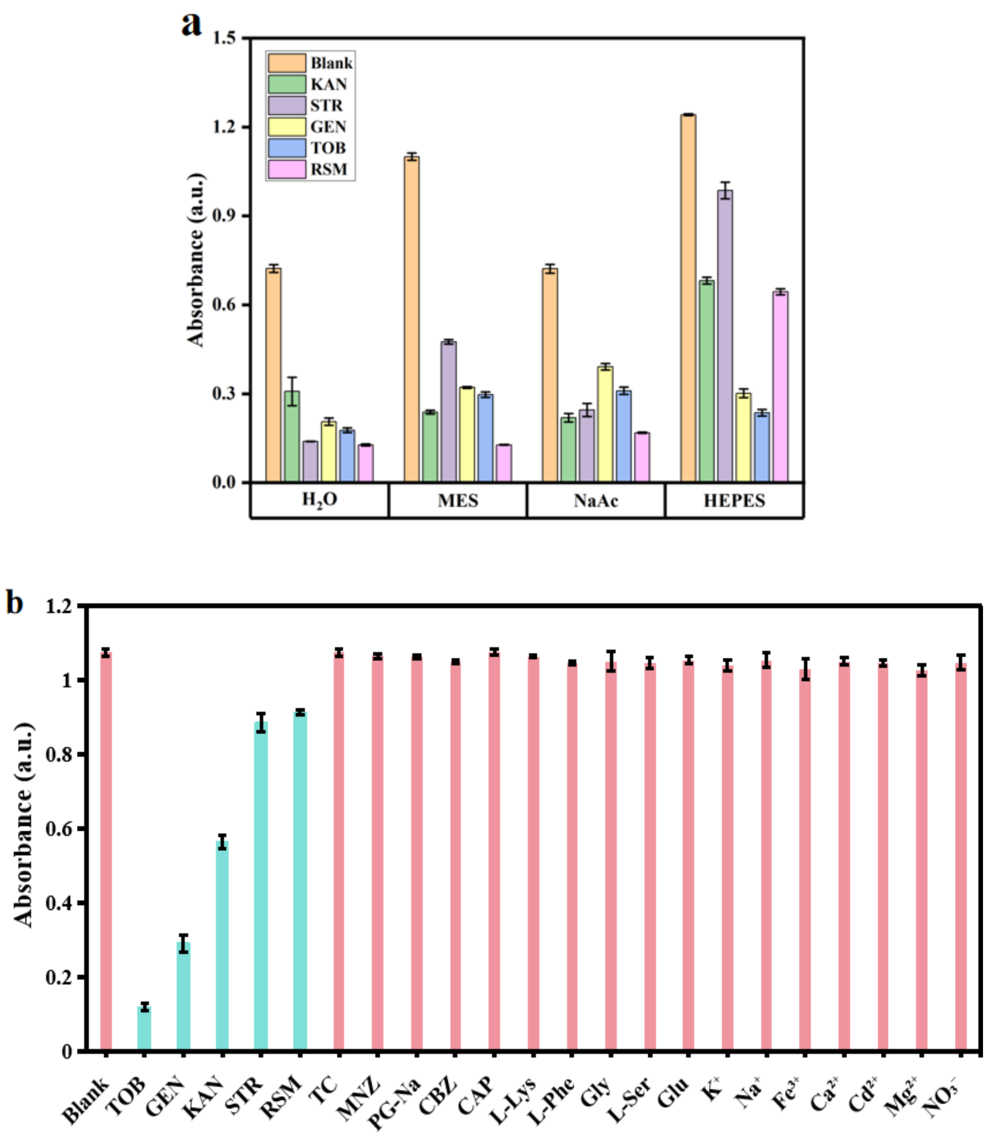

Figure S11. (a) Effect of AGs on laccase-like activity of CH3COOAg in different solutions. (b) Impacts of various interfering substances (1 μM) on laccase-like activity of CH3COOAg.

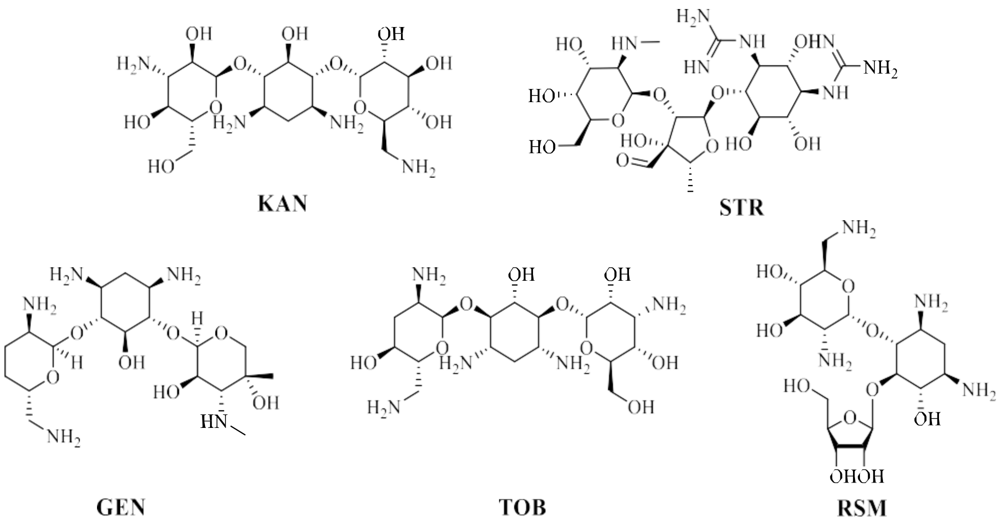

Figure S12. Chemical structures of five AGs.

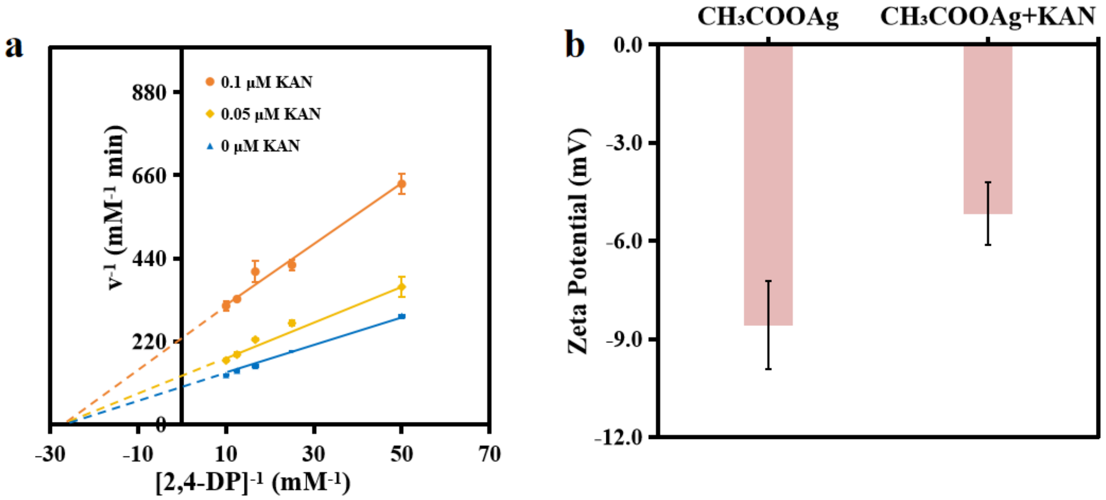

Figure S13. Inhibition mechanism of KAN on CH3COOAg. (a) Lineweaver–Burk plot for oxidation of 2,4-DP catalyzed by CH3COOAg in presence of KAN. (b) Zeta potential of CH3COOAg before and after addition of KAN.

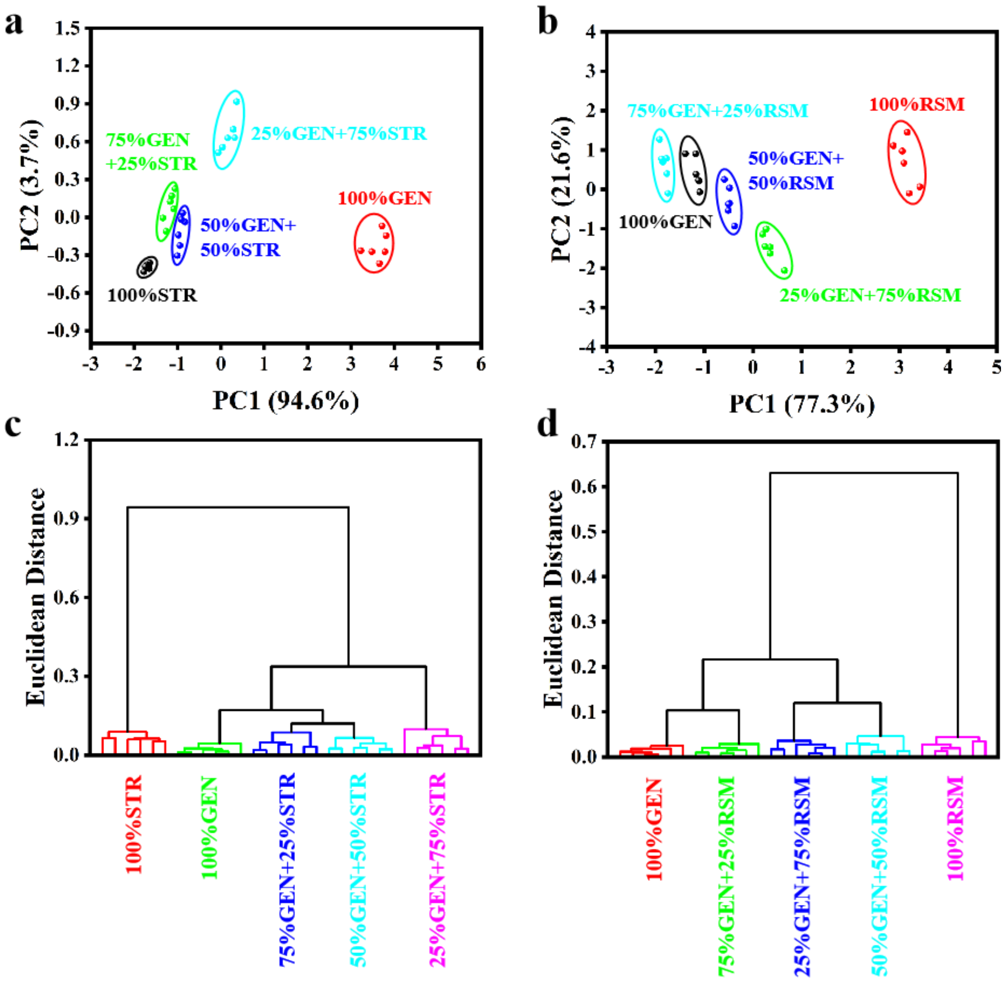

Figure S14. (a: GEN and STR; b: GEN and RSM) PCA diagram for discrimination of two-component AGs; (c: GEN and STR; d: GEN and RSM) HCA diagram for discrimination of two-component AGs.
